# Integrated metabolomic and cytokine profiling reveals biomarkers across the clinical spectrum of lupus nephritis

**DOI:** 10.3389/fimmu.2026.1787160

**Published:** 2026-03-31

**Authors:** Mariana Jiménez-Tirado, Karina Santana-De Anda, Mauricio Arvizu-Hernández, Jennifer Tiaré Balderas-Miranda, Beatriz Alcalá-Carmona, Yatzil Reyna-Juárez, David Eduardo Meza-Sánchez, Nancy Mejía-Domínguez, José Carlos Paez-Franco, Hilda Sánchez-Vidal, Imelda Cecilia Zarzoza-Mendoza, José Luis Maravillas-Montero, José Jiram Torres-Ruiz, Diana Gómez-Martín

**Affiliations:** 1 Departamento de Inmunología y Reumatología, Instituto Nacional de Ciencias Médicas y Nutrición Salvador Zubirán, Mexico City, Mexico; 2 Departamento de Nefrología y Metabolismo Mineral, Instituto Nacional de Ciencias Médicas y Nutrición Salvador Zubirán, Mexico City, Mexico; 3 Escuela de Medicina y Ciencias de la Salud, Instituto Tecnológico y de Estudios Superiores de Monterrey, Mexico City, Mexico; 4 Red de Apoyo a la Investigación, Instituto Nacional de Ciencias Médicas y Nutrición Salvador Zubirán and Universidad Nacional Autónoma de México, Mexico City, Mexico; 5 Departamento de Medicina Molecular y Bioprocesos, Instituto de Biotecnología, Universidad Nacional Autónoma de México, Cuernavaca, Mexico

**Keywords:** systemic lupus erythematosus, lupus nephritis, non-invasive biomarkers, urinary cytokines, metabolomics

## Abstract

**Introduction:**

Lupus nephritis (LN) represents one of the most frequent and severe manifestations of systemic lupus erythematosus (SLE), highlighting an urgent need for novel non-invasive approaches to improve its diagnosis and clinical monitoring.

**Objectives:**

To characterize serum and urinary metabolomic profiles, together with urinary cytokine expression, in patients with lupus nephritis (LN) across the clinical spectrum from active disease, post-induction response, and sustained remission.

**Methods:**

We enrolled 62 patients fulfilling the 2019 ACR/EULAR SLE criteria, divided into three groups: active LN (n=21), post-induction responders at 6 months (n=21), and sustained responders with ≥2 years of remission (n=20). Untargeted metabolomic profiling of serum and urine was performed using GC-MS, and urinary cytokines were measured by luminometry. Statistical analyses included Kruskal-Wallis tests and multinomial logistic regression.

**Results:**

Median age was 36 years; 87% were female. Twelve serum metabolites and ten urinary metabolites differed significantly across groups. Active LN patients showed increased urinary threonate and elevated cytokines (15 out of 17 measured, including IL-10). Serum glycerol was significantly higher in post-induction responders compared to sustained responders, while urinary sucrose was lower in this group. Logistic regression identified urinary threonate as discriminative for active LN, whereas serum glycerol (OR 1.05, 95% CI 1.048–1.050) and urinary sucrose (OR 0.98, 95% CI 0.9802–0.981) differentiated post-induction from sustained response. Increased urinary IL-10 was strongly associated with active LN, while G-CSF discriminated post-induction from sustained responders.

**Discussion and conclusions:**

The combined analysis of serum/urinary metabolomic profiles and urinary cytokines provides discriminatory power to differentiate active LN from remission states, supporting their potential role as non-invasive biomarkers for diagnosis and treatment monitoring.

## Graphical Abstract

**Figure:**
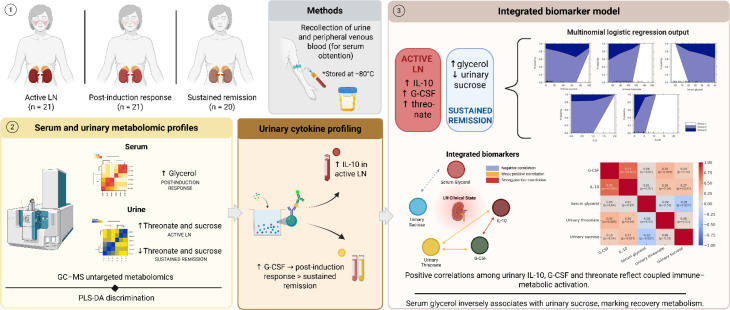
Schematic representation of study design and integrated metabolomic and cytokine profiling findings. (1) Serum and urine samples were collected from patients with active lupus nephritis (n = 21), post-induction responders (n = 21), and patients in sustained remission (n = 20) for untargeted GC–MS metabolomic analysis and urinary cytokine profiling. (2) Active LN was characterized by increased urinary threonate, sucrose, and IL-10 concentrations, whereas post-induction response showed elevated serum glycerol and G-CSF levels. Patients in sustained remission exhibited normalization of these biomarkers. (3) Multinomial logistic regression identified urinary threonate as discriminative for active LN, while serum glycerol and urinary sucrose differentiated post-induction from sustained remission. Correlation analysis revealed coordinated immune–metabolic activation, with positive associations among urinary IL-10, G-CSF, and threonate, and an inverse relationship between serum glycerol and urinary sucrose.

## Introduction

Lupus nephritis (LN) is one of the most severe manifestations of systemic lupus erythematosus (SLE), affecting up to 50% of patients, of whom 5–20% progress to end-stage renal disease requiring renal replacement therapy within 10 years of diagnosis (1). The current diagnostic gold standard is percutaneous renal biopsy, an invasive procedure that carries inherent risks (2). This has fueled interest in non-invasive approaches for the diagnosis and monitoring of LN, among which metabolomic profiling and cytokine measurement in biological samples have emerged as promising strategies (3).

Metabolomic analysis of serum and urine has demonstrated a robust ability to discriminate between patients with active LN, patients with SLE without renal involvement and healthy controls (3). This utility is supported by the metabolic reprogramming of immune effector cells, which rely on aerobic glycolysis to sustain clonal expansion, polarization, and effector functions during disease flares (4). As a result, patients with active LN exhibit characteristic shifts in substrate profiles, including reduced serum amino acid levels, increased serum fatty acids and elevated serum glucose (5, 6). In parallel, multiple urinary cytokines and chemokines have been investigated as auxiliary biomarkers for LN diagnosis and monitoring (7). Beyond their discriminatory capacity between active and inactive LN, these molecules correlate with global disease activity indices, histopathological features and treatment response (8).

Despite this evidence, the combined performance of metabolomic profiling and urinary cytokine analysis across the full clinical spectrum of LN, from active disease to post-induction renal response and sustained remission has not been systematically evaluated. Moreover, data on paired serum and urine metabolomic profiles remain limited. The aim of the present study was to compare serum and urinary metabolomic profiles, integrated with urinary cytokine measurement, in patients with biopsy-proven and/or biochemically active LN versus patients with partial or complete renal response after induction therapy and those with sustained long-term remission.

## Methods

We included adult patients (≥18 years) with a diagnosis of systemic lupus erythematosus (SLE) according to the 2019 ACR/EULAR classification criteria (9), who attended the Instituto Nacional de Ciencias Médicas y Nutrición Salvador Zubirán (INCMNSZ) between June and December 2024 and provided written informed consent. The study was approved by the Research and Ethics Committees of INCMNSZ (reference no. 5082).

Patients were assigned to study groups based on the presence or absence of renal activity, defined by laboratory and/or histopathological criteria. Group 1 comprised patients with active LN, defined as proliferative lupus nephritis (class III or IV), with or without a membranous component (class V), confirmed by renal biopsy or by laboratory criteria including proteinuria >500 mg/day or a urinary protein-to-creatinine ratio (UPCR) >0.5 g/g, an increase in serum creatinine >0.3 mg/dL from baseline and active urinary sediment (leukocyturia >5/HPF, hematuria >5/HPF or granular/erythrocyte casts). All patients also had a SLEDAI-2K score ≥6 (10).

Group 2 included patients with partial or complete renal response 3–6 months after induction therapy. Complete response was defined as UPCR <0.5 g/g, normal creatinine or ≥25% improvement in eGFR if abnormal and bland urinary sediment (11). Partial response was defined as ≥50% reduction in proteinuria with UPCR between 0.5–3 g/g, ≥25% improvement in eGFR if abnormal and bland sediment. The decision to categorize complete and partial responders into a single group reflects the clinical reality of SLE management, where a significant proportion of patients achieve substantial improvement without reaching the criteria for complete remission (12, 13).

Group 3 included patients with complete and sustained renal response for ≥2 years defined as proteinuria <500 mg/day or UPCR <0.5 g/g and inactive urinary sediment (14).

We excluded pregnant or postpartum women, patients with overlap syndromes (except antiphospholipid syndrome and Sjögren’s disease), active or suspected infections (viral, bacterial, parasitic, or fungal), hematologic or solid malignancies and recreational drug use. Patients with class VI LN on biopsy, primary or secondary kidney disease not related to SLE, anuria, renal replacement therapy or prior kidney transplantation were also excluded.

All patients were evaluated by an attending rheumatologist and/or rheumatology fellow, including physical examination and calculation of SLEDAI-2K score at the time of the recruitment. Demographics, disease duration, and metabolic comorbidities such as hypertension, type 2 diabetes, dyslipidemia and obesity (which was assessed through body mass index (BMI), categorized as obesity (BMI >30 kg/m²) or non-obesity) were obtained from the electronic medical record. Laboratory data included lipid profile, 24-hour proteinuria and creatinuria, urinary sediment, complement levels, and autoantibodies (antinuclear antibodies, anti-dsDNA, anti-Sm, antinucleosome and antiphospholipid antibodies). Treatment history was recorded including the type, dose and duration of immunosuppressants, biologics and corticosteroids converted to prednisone equivalent doses. Use of sodium-glucose transport protein 2 (SGLT2) inhibitors, statins, angiotensin-converting enzyme (ACE) inhibitors and angiotensin II receptor blockers (ARBs) were also recorded. In patients with available renal biopsy, histologic class, activity and chronicity indices, as well as percentage of interstitial fibrosis and tubular atrophy were documented. Samples of serum and urine were collected within a 2-to-3-week window of the renal biopsy procedure. For patients in the remission group, histological classification was retrieved from their initial diagnostic biopsy at disease onset, as repeat biopsies are not usually performed for patients who have achieved clinical remission at our institution.

### Metabolomic and cytokine analysis

*1*. Pre-analytical Handling and Sample Collection. All samples were collected under a standardized clinical protocol to minimize technical variability:

- Fasting Status: All participants were under random dietary conditions prior to blood and urine collection.
- Serum Processing: Venous blood (10 ml) was collected in SST tubes. Samples were centrifugated at 3500 revolutions per minute (rpm) for 5 minutes and serum aliquots were immediately transferred to cryovials.
- Urine Collection: Random spot urine samples (10 ml) were collected in sterile containers. Samples were centrifuged at 1600 rpm for 5 minutes to remove cellular debris and supernatant was aliquoted and immediately transferred to cryovials.
- Storage and Stability: All aliquots were stored at -80 °C until analysis. The total storage time before extraction was approximately 6–8 months. To maintain metabolite integrity, no more than one freeze-thaw cycles were permitted.

All samples were analyzed randomly. For serum analysis, 45 µL of serum was mixed with 5 µL of internal standard (IS—tridecanoic acid, 0.1 mg/mL) in 150 µL of 1:3 chloroform–methanol and thoroughly vortexed for 2 min. For urine samples, 50 µL was mixed with 5 µL of IS and 300 µL of pure methanol. Both types of samples were incubated at -20 °C for 20 min and then centrifuged at 14,000 rpm for 15 min. The recovered supernatants were dried overnight using a SpeedVac system (Eppendorf). Each dried precipitate was redissolved in 25 µL of methoxyamine hydrochloride solution and incubated for 90 min at 37 °C in a shaking incubator. Subsequently, 50 µL of MSTFA + 1% TMCS was added to each sample, followed by incubation for 30 min at 37 °C. One microliter of the derivatized sample was injected into a GC/MS system (Agilent 5977A/7890B, Santa Clara, CA, USA) equipped with an automatic autosampler (G4513A, Agilent) and analyzed under the following conditions: splitless mode with a column flow of 1 mL/min, inlet temperature 200 °C, EI source temperature 200 °C, and interface temperature 250 °C. An HP-5ms column (30 m × 250 µm × 0.25 µm, Agilent) was used with helium (99.9999% purity) as the carrier gas. The oven temperature program started with a 1-min hold at 60 °C, followed by a ramp of 10 °C/min to 325 °C, and a final hold of 10 min.

To monitor system suitability and analytical stability throughout the runs, a quality control (QC) pooled sample—prepared by combining equal volumes of all serum or urine samples—was injected every four samples. Metabolites exhibiting a relative standard deviation (RSD) >30% in the QC samples were discarded, in line with Fiehn Laboratory recommendations (15). Tight clustering of QC samples in principal component analysis (PCA) confirmed the reliability and consistency of the metabolic profiles across the entire analysis (**Supplementary Figure 1**). For data processing, GC/MS raw data were converted to.mzData format using Agilent ChemStation software. Feature detection, spectral deconvolution, and peak alignment were performed using MZmine 2.0 software with the following parameters: retention time range 5.5–27.5 min; m/z range 50–500; m/z tolerance 0.5 Da; noise level 1 × 10³; and peak duration range 0.01–0.2 min. Additional metabolite filtering followed the “80% rule,” retaining features present in at least 80% of samples in at least one group. Identifications were performed by matching against the National Institute of Standards and Technology (NIST) 2.0 spectral library. Matches with a similarity score >700 (R > 70%) were accepted as reliable identifications; lower scores were classified as unknowns and excluded. For statistical analysis, Metaboanalyst 4.0 was employed. For serum and urine data, raw peak heights were normalized by sum for univariate analyses. For multivariate analyses, data were sum-normalized, log-transformed, and autoscaled. Sum normalization was preferred for urine samples, as it has been shown to yield valid results than normalization based on urine total volume, osmolality, or creatinine levels (16–18). Cytokine urinary levels were assessed using the Bio-Plex Pro Human Cytokine Screening Panel 17 Plex kit (Bio-Rad, CA, USA). Reading was performed using the Luminex platform (Bio-Rad, CA, USA) and results were expressed in pg/mL.

### Statistical analysis

Sample size was determined *a priori* to detect significant differences in metabolite concentrations based on previous reports for detecting differences in serum choline levels between LN LN (1.00, IQR 0.37-1.36) versus SLE patients without renal involvement (0.61, IQR 0.09-0.96) (5) to achieve 80% power with a 5% alpha error, a minimum of 15 patients per group was required. Our inclusion of approximately 20 patients per group ensures sufficient statistical power for the reported signatures.

Categorical variables were expressed as frequencies and percentages; continuous variables as medians and interquartile ranges (IQR). Comparisons were performed using Kruskal–Wallis tests for continuous variables and Chi-square for categorical variables. Principal component analysis (PCA) was used to visualize group variation in metabolomic data, and differential metabolites were identified using partial least squares discriminant analysis (PLS-DA). Model validation was performed by variable relevance in projection (VIP >1) and permutation tests, with discriminant metabolites selected at p<0.05. Univariate analyses were also performed. To minimize the risk of false-positive results, p-values from univariate analyses (Kruskal-Wallis) were adjusted using the False Discovery Rate (FDR) procedure. Significant variables were included in a multinomial logistic regression model from which probability plots were constructed to visualize the predicted likelihood of each group as a function of metabolite and cytokine concentrations. Spearman’s rank correlation analyses were conducted to explore the relationships between metabolites and urinary cytokines that were identified as significant predictors in the multinomial logistic regression model. All analyses were conducted using R software, with p<0.05 considered statistically significant.

## Results

Urine and serum samples were obtained from 21, 21, and 20 patients in groups 1, 2, and 3, respectively. As shown in **Table 1**, the study population included adults with a median age of 36 years of whom 87% were female. It should be noted that disease duration was significantly longer in Group 3 compared to the other groups. This difference is inherent to the clinical definition of the group itself as patients were required to demonstrate sustained clinical and biochemical stability for a minimum of two consecutive years without relapses. Therefore, a longer disease course was a prerequisite for inclusion in this clinical group.

**Table 1:** Demographic and clinical characteristics of the study population according to study groups.

Variables	Active LN Group 1 (n=21)	Post-induction responders Group 2 (n=21)	Sustained responders Group 3 (n=20)	P-value
Age (years) *median (IQR)*	35 (30, 43)	34 (30, 44)	44 (35, 50)	0.077
Female *n (%)*	17 (81%)	19 (90%)	18 (90%)	0.7
Type 2 diabetes *n (%)*	2 (9.5%)	1 (4.8%)	1 (5%)	>0.9
Hypertension *n (%)*	3 (14%)	3 (14%)	1 (5%)	0.7
Dyslipidemia *n (%)*	1 (4.8%)	1 (4.8%)	0 (0%)	>0.9
Obesity *n (%)*	4 (19%)	5 (24%)	6 (30%)	0.7
Disease duration (months) *median (IQR)*	132 (108, 156)	120 (48, 156)	186 (126, 231)	0.045
SLEDAI-2K *median (IQR)*	16 (10, 18)	4 (4, 6)	2 (0, 4)	<0.001
Constitutional *n (%)*	2 (9.5%)	0 (0%)	0 (0%)	0.3
Mucocutaneous *n (%)*	7 (33%)	0 (0%)	0 (0%)	<0.001
Hematologic *n (%)*	2 (9.5%)	0 (0%)	0 (0%)	0.3
Serosal *n (%)*	1 (4.8%)	0 (0%)	0 (0%)	>0.9
Neuropsychiatric *n (%)*	1 (4.8%)	0 (0%)	0 (0%)	>0.9
Articular *n (%)*	2 (9.5%)	0 (0%)	0 (0%)	0.3

IQR, interquartile range; SLEDAI-2K, Systemic Lupus Erythematosus Disease Activity Index 2000.

No differences were observed in the prevalence of metabolic comorbidities. Median SLEDAI-2K score was significantly higher (16 points, IQR 10-18) in the active LN group compared with patients in post-induction and sustained response. A higher proportion of group 1 patients exhibited mucocutaneous activity at enrolment (33%) than group 2 and 3 with no difference across other clinical domains.

Patients with active LN had subnephrotic proteinuria (median 2.2 grams per day, IQR 1.3-3.4). This group also showed significantly higher C-reactive protein levels and neutrophil-to-lymphocyte ratio, as well as higher serum concentrations of total cholesterol, LDL cholesterol, and triglycerides. Conversely, they had lower mean hemoglobin and serum albumin. Estimated glomerular filtration rate did not differ among groups. In group 2, 13 patients (62%) achieved complete renal response while 8 (38%) were classified as partial responders. Regarding immunological variables, active LN patients exhibited lower complement levels and higher median anti-dsDNA antibody titers (209 U/mL, IQR 71- 308), without differences in anti-Sm, antinucleosome or antiphospholipid antibodies (**Table 2**).

**Table 2:** Laboratory features according to each study group.

Variables	Active LN Group 1 (n=21)	Post-induction responders Group 2 (n=21)	Sustained responders Group 3 (n=20)	P-value
Hemoglobin (g/dl) *median (IQR)*	12.2 (10.3, 13.2)	13.3 (12, 14.3)	13.9 (13.2, 14.1)	0.005
Total leukocytes (cells/mm³) *median (IQR)*	6.5 (5.4, 8.3)	6.9 (4.5, 7.5)	6.0 (4.8, 6.6)	0.4
Total neutrophils (cells/mm³) *median (IQR)*	4.6 (3.6, 5.8)	4.2 (3.1, 4.8)	3.1 (2.5, 3.5)	0.018
Total lymphocytes (cells/mm³) *median (IQR)*	1.2 (0.95, 1.7)	1.4 (1.0, 1.9)	1.6 (1.4, 2.1)	0.2
Platelets (cells/mm³) *median (IQR)*	305 (189, 351)	292 (234, 308)	261 (235, 310)	>0.9
Neutrophil-to-lymphocyte ratio *median (IQR)*	3.40 (2.64, 4.80)	2.70 (2.10, 3.40)	1.89 (1.40, 2.35)	0.002
CRP (mg/l) *median (IQR)*	0.50 (0.11, 2.06)	0.12 (0.05, 0.22)	0.18 (0.10, 0.31)	0.025
Albumin (g/dL) *median (IQR)*	3.15 (2.62, 3.41)	3.99 (3.68, 4.06)	4.16 (4.04, 4.33)	<0.001
Globulins (g/dL) *median (IQR)*	2.65 (2.50, 3.08)	2.30 (2.14, 2.78)	3.02 (2.66, 3.22)	0.001
Total cholesterol (mg/dL) *median (IQR)*	191 (156, 244)	152 (133, 204)	140 (127, 166)	0.002
LDL cholesterol (mg/dL) *median (IQR)*	119 (91, 149)	88 (63, 123)	79 (66, 111)	0.006
HDL cholesterol (mg/dL) *median (IQR)*	46 (37, 51)	57 (45, 63)	47 (43, 53)	0.085
Triglycerides (mg/dL) *median (IQR)*	156 (126, 190)	106 (81, 136)	89 (81, 131)	0.001
eGFR (ml/min/1.73 m²) *median (IQR)*	103 (86, 118)	113 (94, 129)	107 (99, 115)	0.4
Proteinuria (gr/24 hours) *median (IQR)*	2.2 (1.3, 3.4)	0.215 (0.140, 0.402)	0.139 (0.098, 0.294)	<0.001
Anti-dsDNA titers (U/mL) *median (IQR)*	209 (71, 308)	67 (26, 255)	18 (12, 39)	0.001
Anti-Sm titers (U/mL) *median (IQR)*	48 (8, 126)	8 (7, 37)	16 (10, 171)	0.5
Antinucleosome titers (U/mL) *median (IQR)*	143 (57, 261)	110 (25, 224)	46 (12, 132)	0.3
Positive APS serology *n (%)*	6 (29%)	2 (9.5%)	6 (30%)	0.2
Complement C3 (mg/dL) *median (IQR)*	71 (58, 91)	105 (83, 117)	116 (102, 137)	<0.001
Complement C4 (mg/dL) *median (IQR)*	8 (8, 18)	16 (11, 23)	19 (10, 25)	0.022
Renal biopsy* n (%)	14 (67%)	17 (81%)	11 (55%)	0.2

*Renal biopsy performed at time of diagnosis of LN flare. CPR, C-reactive protein; eGFR, estimated glomerular filtration rate; APS, antiphospholipid syndrome.

Significant differences were observed between groups regarding pharmacological management, specifically for mycophenolate mofetil (p=0.006), and SGLT2 inhibitors (p=0.028), corticosteroid doses (p=0.002), although the proportion of individuals on high-dose prednisone (defined as >0.5 mg/kg/day) did not differ between groups as detailed in **Supplementary Table 1**. Post-induction responders received tacrolimus more often, while none received azathioprine. Patients with sustained response were treated with lower average doses of mycophenolate mofetil and more frequently with azathioprine. No significant differences were found in the use of other immunosuppressants, lipid-lowering agents, ACE inhibitors or ARBs. These differences align with standard clinical practice, where treatment intensity is adjusted according to renal activity.

Prior to multivariate modeling, an unsupervised Principal Component Analysis (PCA) was performed to assess the technical quality of the metabolomic runs. The PCA scores plots (**Supplementary Figure 1**) showed that the pooled quality control (QC) samples clustered tightly in the center of the distribution for both serum and urine datasets. This high reproducibility indicates minimal analytical drift and confirms that the observed metabolic variations are of biological origin rather than technical artifacts. Furthermore, PCA revealed a consistent overlap between clinical groups, justifying the subsequent use of supervised models (PLS-DA) to identify specific discriminatory features.

Regarding the robustness of our PLS-DA models, we observed distinct performances between the two biofluids. The serum metabolomic model demonstrated statistical significance through permutation testing (p = 0.016, 16/1000 permutations) and achieved a cross-validation Q2 of 0.209 for the first component. VIP scores identified key metabolites driving this separation, including tryptophan, α-ketoglutarate, myo-inositol, and erythritol (**Supplementary Figure 2**). In contrast, the urinary model was less robust and showed a trend towards significance (p = 0.09). Consequently, urinary findings were treated as exploratory, focusing on the identification of individual candidate metabolites with high VIP scores, such as threonic acid which remained robust in univariate analyses (**Supplementary Figure 3**). Detailed model validation metrics, including R2, Q2, and permutation results, are provided in **Supplementary Figures 2**, **3**.

**Figure 1:**
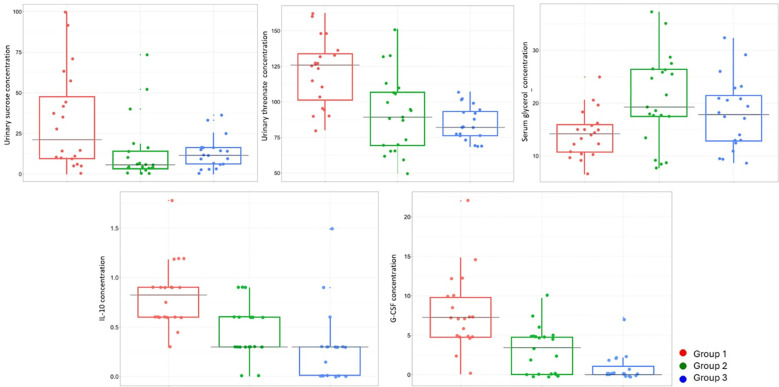
Box plots of discriminatory metabolites and cytokines across study groups. Box plots illustrating the maximum peak heights (sum-normalized) of urinary sucrose, urinary threonate, serum glycerol, IL-10, and G-CSF concentrations among the three study groups. Group 1: patients with active lupus nephritis, Group 2: patients with partial or complete renal response after induction therapy, and Group 3: patients with sustained complete renal response. Boxes represent the interquartile range (IQR), horizontal lines indicate the median, and whiskers denote minimum and maximum values within 1.5 × IQR.

Univariate analysis of normalized metabolite concentrations revealed significant differences in 12 serum and 10 urinary metabolites (**Supplementary Tables 2**, **3**). Urinary cytokine concentrations were significantly higher in active LN, except for IL-4 and IL-12 which were virtually undetectable in all groups (**Supplementary Table 4**). As shown in the box plot analysis (**Figure 1**), urinary sucrose and urinary threonate concentrations were significantly higher in Group 1 compared with Groups 2 and 3. Conversely, serum glycerol levels were elevated in Group 2 relative to the other groups. Regarding cytokines, IL-10 and G-CSF showed a marked reduction in Groups 2 and 3 compared with Group 1. There was no correlation between histopathologic findings at time of diagnosis in the active LN group with serum and urine metabolomic and urinary profile findings.

Multinomial logistic regression (**Table 3**) identified urinary threonate as discriminative across groups, while serum glycerol (OR 1.05, 95% CI 1.048–1.050) and urinary sucrose (OR 0.98, 95% CI 0.9802–0.981) differentiated post-induction responders from sustained responders. Among urinary cytokines, IL-10 was strongly associated with active LN (OR 1.25x10^-12^, 95% CI 2.02x10^-17^–7.76x10^-08^), whereas G-CSF discriminated between groups 2 and 3 (OR 0.5538, 95% CI 0.5517–0.5559). Odds ratios for IL-10 are reported in exponential notation due to the high sensitivity of the multinomial model due to the raw concentration scales; these values indicate a strong and significant association with the clinical group.

**Table 3:** Multinomial logistic regression results for discriminative biomarkers among renal activity groups.

Metabolite/ urinary cytokine	Group 1 vs 2 OR (95% CI)	Group 2 vs 3 OR (95% CI)	Group 1 vs 3 OR (95% CI)
Urinary sucrose	NS	0.9809 (0.9802–0.981) β = 0.01 (0.018-0.02)	NS
Urinary threonate	3.0 (1.56–5.77) β =-1.1 (-1.7 to-0.44)	1.027 (1.026-1.0274) β =-0.02 (-0.027 to -0.026)	3.09 (1.6-5.9) β =-1.12 (-1.78 to -0.47)
Serum glycerol	NS	1.049 (1.048–1.05) β =20.6 (20.18-21.05)	NS
IL-10	1.25x10^-12^ (2.02x10^-17^–7.76x10^-08^) β =-27.4 (-38.4 to -16.37)	0.9354 (0.8913–0.9817) β =-0.06 (-0.11 to -0.018)	1.17x10^-12^ (1.80x10^-17^–7.62x10^-08^) β =-27 (-28.5 to -16.39)
G-CSF	NS	0.5538 (0.5517–0.5559) β =-0.590 (-0.594 to-0.58)	NS

Group 1 = active lupus nephritis, group 2= post-induction responders, group 3= sustained remission. NS, Not significant; G-CSF, granulocyte colony-stimulating factor.

**Figure 2:**
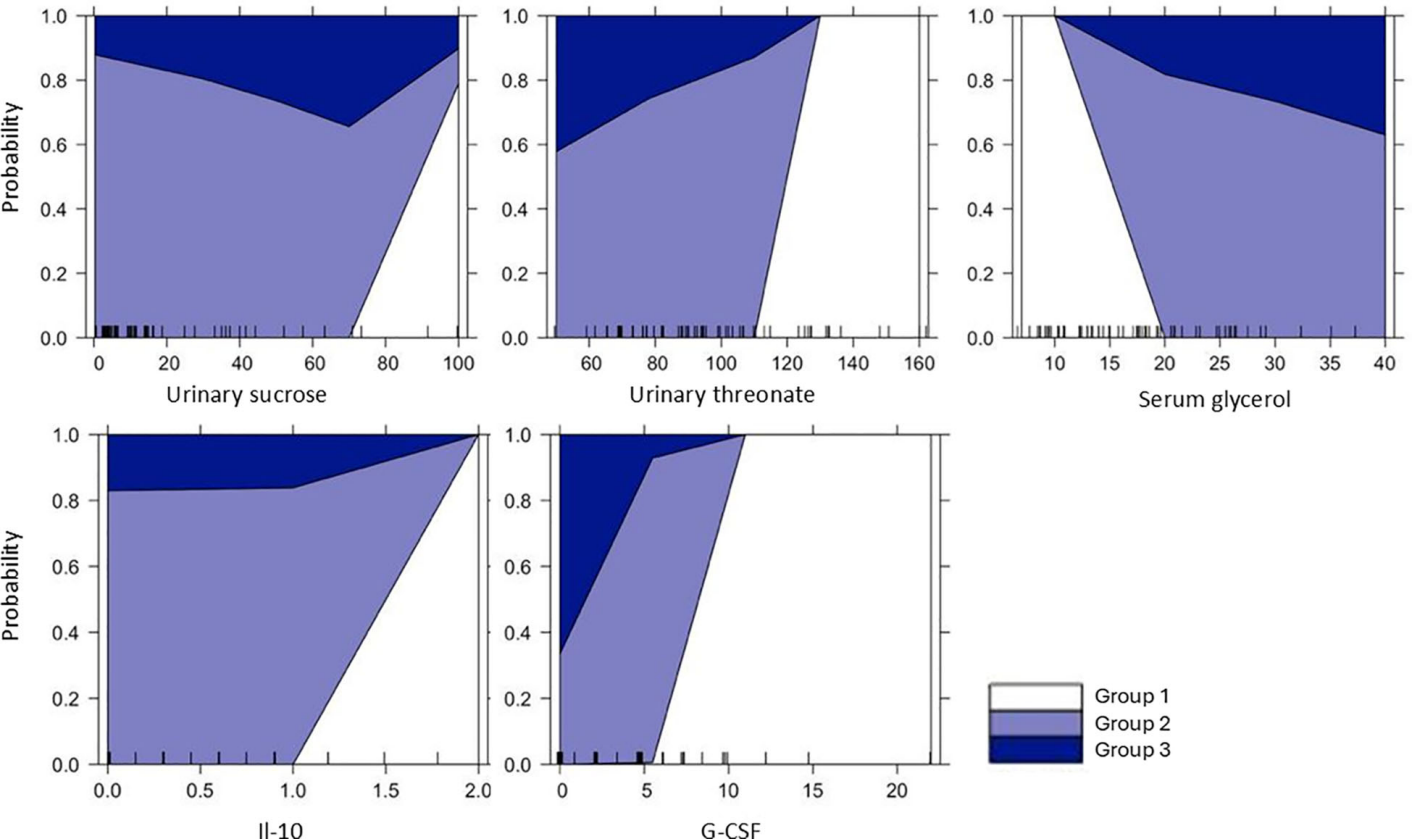
Modeled group probabilities based on urinary metabolites, serum glycerol, and urinary cytokines. Multinomial logistic regression probability plots for urinary sucrose, urinary threonate, serum glycerol, IL-10, and G-CSF across lupus nephritis groups. The plots display predicted group membership probabilities as a function of metabolite or cytokine levels, illustrating their discriminative value amongst patient groups. Group 1: patients with active lupus nephritis, Group 2: patients with partial or complete renal response after induction therapy, and Group 3: patients with sustained complete renal response.

Probability plots demonstrated that selected metabolites and cytokines displayed differential discriminative capacity across the three lupus nephritis groups (**Figure 2**). The probability of belonging to the active LN group increased as urinary levels of sucrose and threonate were higher. Serum glycerol exhibited a pattern where higher concentrations were linked to increased probability of belonging to the sustained response group (Group 3). Among cytokines, higher levels of IL-10 and G-CSF predicted greater probability of active disease.

**Figure 3:**
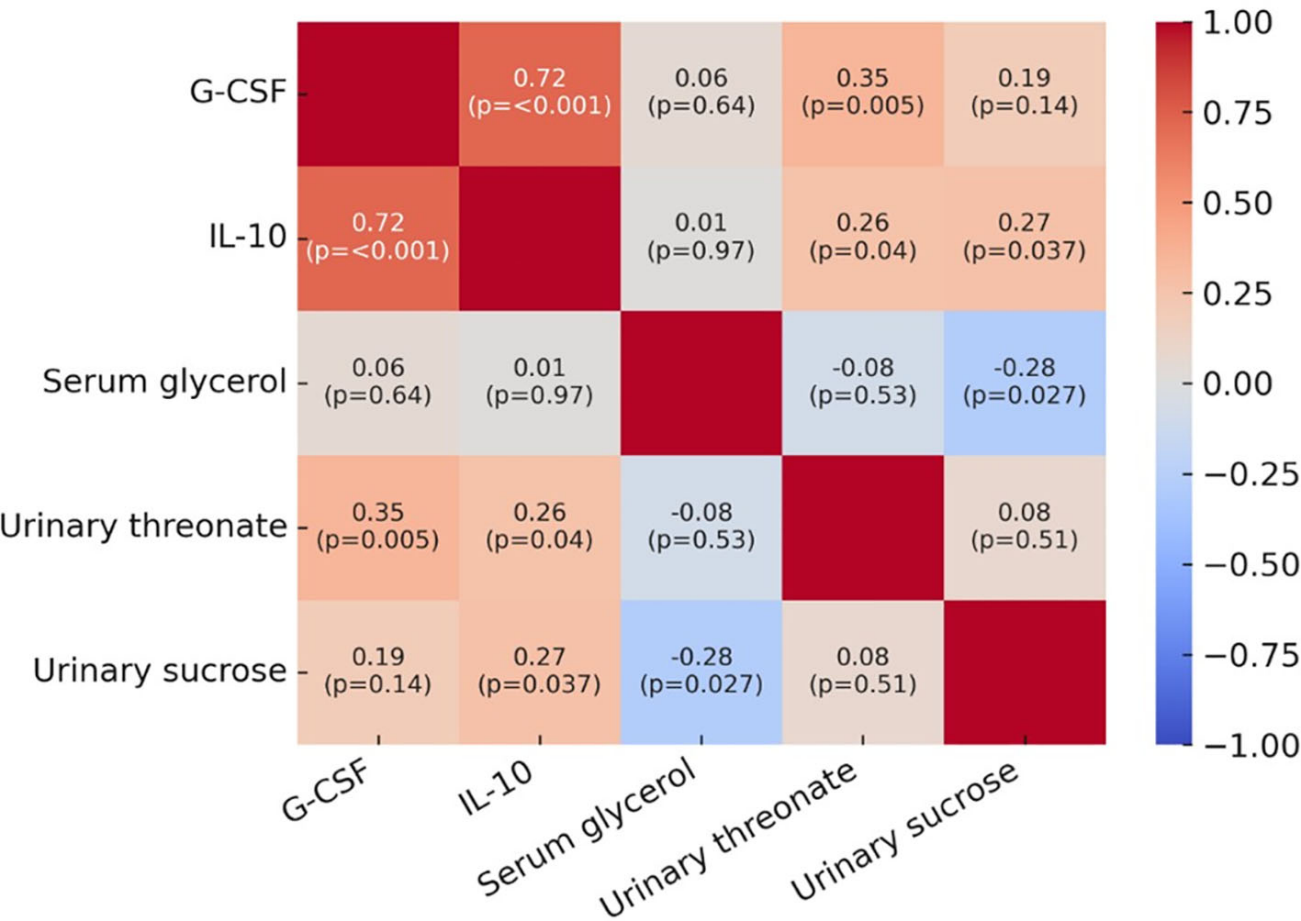
Correlation matrix between serum/urinary metabolites and urinary cytokines. Spearman’s correlation heatmap between key metabolites and cytokines. The analysis included serum glycerol, urinary threonate, urinary sucrose, G-CSF, and IL-10. Strong positive correlations were observed between G-CSF and IL-10 (ρ = 0.72, p < 0.001), while moderate correlations were found between urinary threonate and both G-CSF (ρ = 0.35, p = 0.005) and IL-10 (ρ = 0.26, p = 0.04). Serum glycerol showed a significant negative correlation with urinary sucrose (ρ = –0.28, p = 0.027). Color intensity represents the strength and direction of correlations (red = positive, blue = negative).

Spearman’s correlation analysis revealed significant associations between cytokines and metabolites (**Figure 3**). G-CSF showed a strong positive correlation with IL-10 (ρ = 0.72, p < 0.001) and a moderate correlation with urinary threonate (ρ = 0.35, p = 0.005). IL-10 was also positively correlated with urinary sucrose (ρ = 0.27, p = 0.037) and urinary threonate (ρ = 0.26, p = 0.04). Conversely, serum glycerol displayed a negative correlation with urinary sucrose (ρ = –0.28, p = 0.027). These findings suggest coordinated regulation between systemic metabolic changes and local immune responses in lupus nephritis.

## Discussion

Our study identified metabolites not previously described in the literature as discriminative among the clinical spectrum of lupus nephritis, providing a broader view of the complex metabolic processes altered in this disease, as well as their interaction with urinary cytokines that exhibit dual roles in inflammatory pathways. Glycerol is a key intermediate of lipid metabolism released from adipose tissue during lipolysis and can be used as a substrate for hepatic gluconeogenesis (19). In patients with LN, alterations in lipids and their derivatives have been identified in serum (6, 20). In our study, the difference in serum glycerol concentration appeared to be more relevant in discriminating between patients with LN who responded to induction therapy and those with sustained renal response. The observed variation in glycerol may be related to an increased demand for this energy substrate to support hepatic gluconeogenesis. Similarly, in a study of over 1,000 participants with diabetic nephropathy, a decrease in glycerol-3-galactoside, a glycerol precursor, was associated with estimated glomerular filtration rate (21), highlighting alterations in glycerolipid metabolism as markers of renal disease regardless of autoimmune etiology, highlighting its role in renal tissue damage.

Threonic acid, an oxidative metabolite of ascorbic acid that may also originate from the pentose phosphate pathway (22), showed higher concentrations in the urine and serum of patients with active LN. Organic acids, the chemical class to which threonic acid belongs, have been reported to distinguish LN from SLE without renal activity; however, this observation had not been specifically made for threonic acid (23). Urinary threonic acid has also been reported to increase in renal dysplasia in pediatric patients, a condition characterized by disturbances in fatty acid, amino acid, and purine oxidation within the renal parenchyma, processes that may be related to interstitial fibrosis or early stages of chronic kidney disease (24). Interestingly, elevated threonic acid concentrations in cerebrospinal fluid metabolomic profiling of Parkinson’s disease suggested that increased levels of this ascorbic acid degradation product represent a disturbance in the oxidative stress response (25). Additionally, in an animal model, threonic acid was identified as a differential urinary metabolite for detecting renal injury secondary to NSAID use (26). Therefore, increased threonic acid in body fluids may reflect reduced antioxidant capacity of ascorbic acid across multiple pathological conditions, including LN. These findings highlight the importance of metabolic and acid-base disturbances in LN and suggest potential avenues for biomarker development and therapeutic response monitoring.

Sucrose is a dietary disaccharide composed of glucose and fructose that is normally absent from urine. Urinary sucrose has been described as a biomarker of total dietary sucrose intake, correlating with anthropometric markers such as waist circumference and waist-to-hip ratio (27).

Regarding urinary cytokines, it is important to highlight the complex roles that IL-10 and G-CSF play in LN. Despite their classic anti-inflammatory role, the observed increase in active LN may reflect heightened B-cell hyperactivity and overall inflammation, rather than successful regulation of the immune response. IL-10 has emerged as a discriminative biomarker in patients with active LN. Quantitative studies have demonstrated that urinary IL-10 levels are significantly elevated in patients with active LN compared to those with hypertensive nephrosclerosis and healthy controls. IL-10 plays a dual role in lupus nephritis, being essential for limiting renal inflammation through suppression of Th1 responses and inhibition of proinflammatory cytokine production (28). However, IL-10 also promotes the differentiation of activated B cells into antibody-producing plasma cells, particularly through extrafollicular mechanisms. This proinflammatory effect has been observed in both murine and human lupus, where elevated IL-10 levels correlate with disease activity and nephritis severity (29, 30), in agreement with our results, IL-10 concentrations were elevated during active LN and decreased after induction therapy, suggesting its potential use as a biomarker to monitor disease activity and therapeutic response.

Granulocyte colony-stimulating factor (G-CSF) plays a multifaceted role in LN pathophysiology, modulating both innate and adaptive immune responses. In murine lupus models, G-CSF administration induces the expansion of regulatory T cells (Tregs) and myeloid-derived suppressor cells (MDSCs), leading to reduced proteinuria, lower anti-dsDNA autoantibodies, improved renal function, and attenuation of renal histopathologic damage. This protective effect is mainly mediated by decreased infiltration of inflammatory cells (T and B lymphocytes, granulocytes, and monocytes) into the kidney and reduced local production of proinflammatory cytokines such as MCP-1, IL-6, IL-2, TNF-α, and IL-17 (31). However, the effect of G-CSF may be dose-dependent, as low doses have been shown to promote a Th2 profile and increase immune complex deposition in glomeruli (32). In our study, elevated G-CSF levels during active disease and following induction therapy may reflect adaptive responses to the inflammatory milieu in LN.

This study analyses paired serum and urine metabolomic profiles together with urinary cytokine analysis in patients with LN at different disease stages, illustrating metabolite–cytokine axes underlying disease activity and treatment response. The use of GC-MS provided high-sensitivity profiling of small, polar metabolites central to energy metabolism, such as organic acids and sugars (15). However, we acknowledge that this platform is less suited for the analysis of intact complex lipids compared to LC-MS/MS (33). Consequently, our findings primarily reflect alterations in the TCA cycle and carbohydrate pathways, while potential lipidomic signatures of LN may remain underrepresented. Future studies using multi-platform approaches could broaden the molecular coverage (34).

Our study however has further limitations that should be acknowledged. First, its cross-sectional design precludes the establishment of definitive causal relationships, therefore longitudinal studies are required to confirm the predictive value of these biomarkers for long-term renal outcomes. Second, while clinical comorbidities such as obesity (BMI >30 kg/m²) were balanced across groups suggesting that the identified metabolic signatures are primarily driven by the immunopathological state of lupus nephritis rather than underlying adiposity or metabolic syndrome we did not incorporate formal insulin resistance assessments (such as HOMA-IR scoring) to further refine these associations. Additionally, dietary intake was not strictly controlled, therefore the presence of sucrose and threonate as potential biomarkers requires caution, as these molecules can be derived from dietary intake or supplements. While no vitamin supplementation was used by the patients in our study, the potential influence of xenobiotics cannot be entirely ruled out. Future validation studies should incorporate strict dietary standardization to confirm the disease-specificity of these metabolic signatures.

Finally, our focus was discriminating between distinct clinical stages within the spectrum of lupus nephritis, rather than providing a diagnostic tool against healthy individuals; future studies including non-renal SLE and healthy controls will be necessary to further establish the absolute specificity of these biomarkers and establish reference thresholds with diagnostic potential.

We acknowledge the difference in disease duration between our study groups, particularly the longer duration in the sustained remission group as this difference in duration is a direct consequence of our stringent inclusion criteria for clinical stability. We hypothesize that the metabolic and cytokine profiles identified in Group 3 may not be a byproduct of chronicity, but rather a reflection of an immunometabolic state of stability that characterizes long-term remission. Future longitudinal studies could further dissect the influence of time-to-remission on the metabolic signature.

A key consideration in clinical metabolomics is the potential confounding effect of pharmacological treatment. In our cohort, patients with higher disease activity (Group 1) received more intensive immunosuppression compared to those in sustained remission (Group 3). We contend that adjusting for these medications as independent covariates would introduce ‘over-adjustment’ bias, as treatment intensity serves as a direct clinical proxy for the disease activity under investigation. Furthermore, while the use of SGLT2 inhibitors was higher in Group 2, the total number of users was low (n=12), limiting their impact on the global metabolic signature. Thus, the profiles reported here reflect the ‘real-world’ immunometabolic spectrum of LN, where pharmacological intervention is an inherent and inseparable component of the patient’s clinical state.

In conclusion, our study provides an integrated metabolomic analysis of the clinical spectrum of lupus nephritis. While these results are hypothesis-generating and require validation in external, independent cohorts, the identified signatures provide a potential non-invasive tool for monitoring disease activity and response to treatment.

## Data Availability

The raw data supporting the conclusions of this article will be made available by the authors, without undue reservation.

## Supplementary material

Supplementary Table 1 Therapeutic variables according to study group. MMF, mycophenolate mofetil; ACE, angiotensin-converting enzyme; ARBs, angiotensin II receptor blockers; SGLT2, sodium-glucose transport protein 2 (SGLT2) inhibitors.

Supplementary Table 2 Serum metabolomic profile according to study group

Supplementary Table 3 Urinary metabolomic profile according to study group. ^++^ Different retention time.

Supplementary Table 4 Urine cytokines according to study groups. G-CSF, Granulocyte colony-stimulating factor; GM-CSF, Granulocyte-macrophage colony-stimulating factor; IFN-γ, Interferon-gamma; MCP-1, Monocyte chemoattractant protein-1; MIP-1b, Active Macrophage Inflammatory Protein 1 Beta; TNF-α, Tumor necrosis factor-alpha. FDR; False discovery rate.
