# Supplementary figures and images for: Integrated metabolomic and cytokine profiling reveals biomarkers across the clinical spectrum of lupus nephritis
Source: Front Immunol. 2026 Mar 31;17:1787160. doi: 10.3389/fimmu.2026.1787160 (PMC13076570; doi:10.3389/fimmu.2026.1787160)

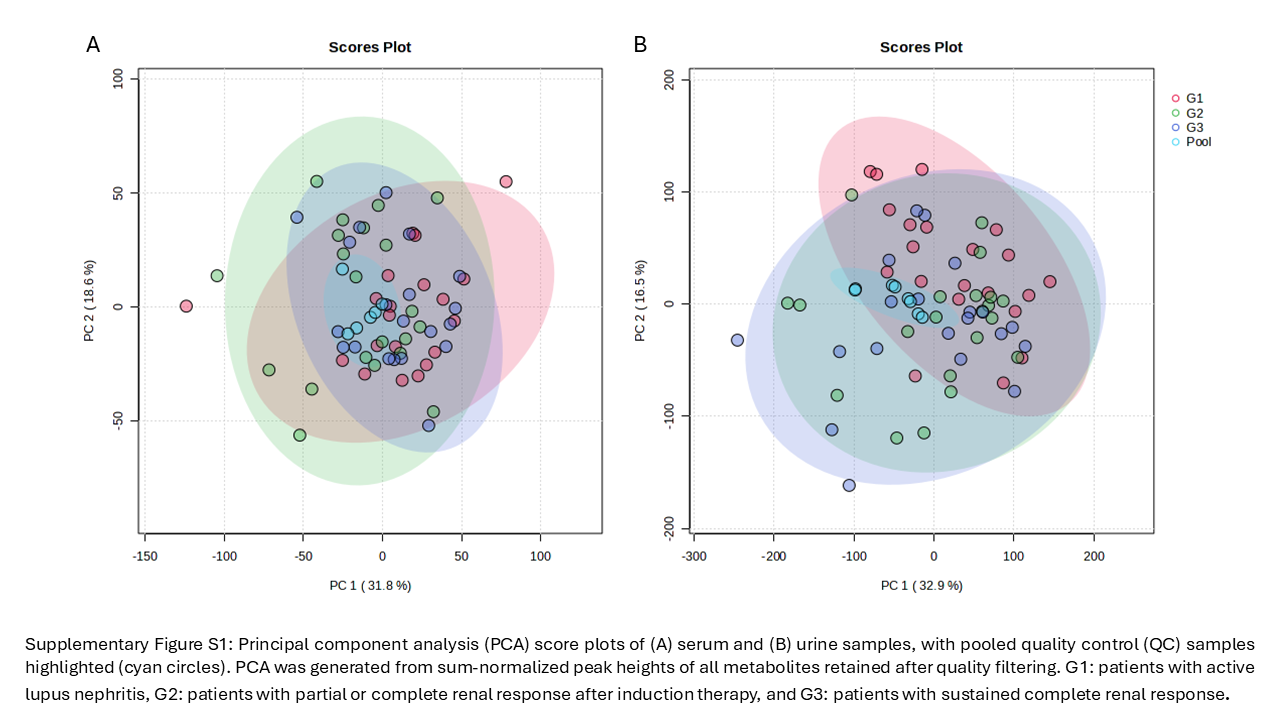

Supplement: Supplementary Table 1 — Therapeutic variables according to study group. MMF, mycophenolate mofetil; ACE, angiotensin-converting enzyme; ARBs, angiotensin II receptor blockers; SGLT2, sodium-glucose transport protein 2 (SGLT2) inhibitors. [file Image1.png]

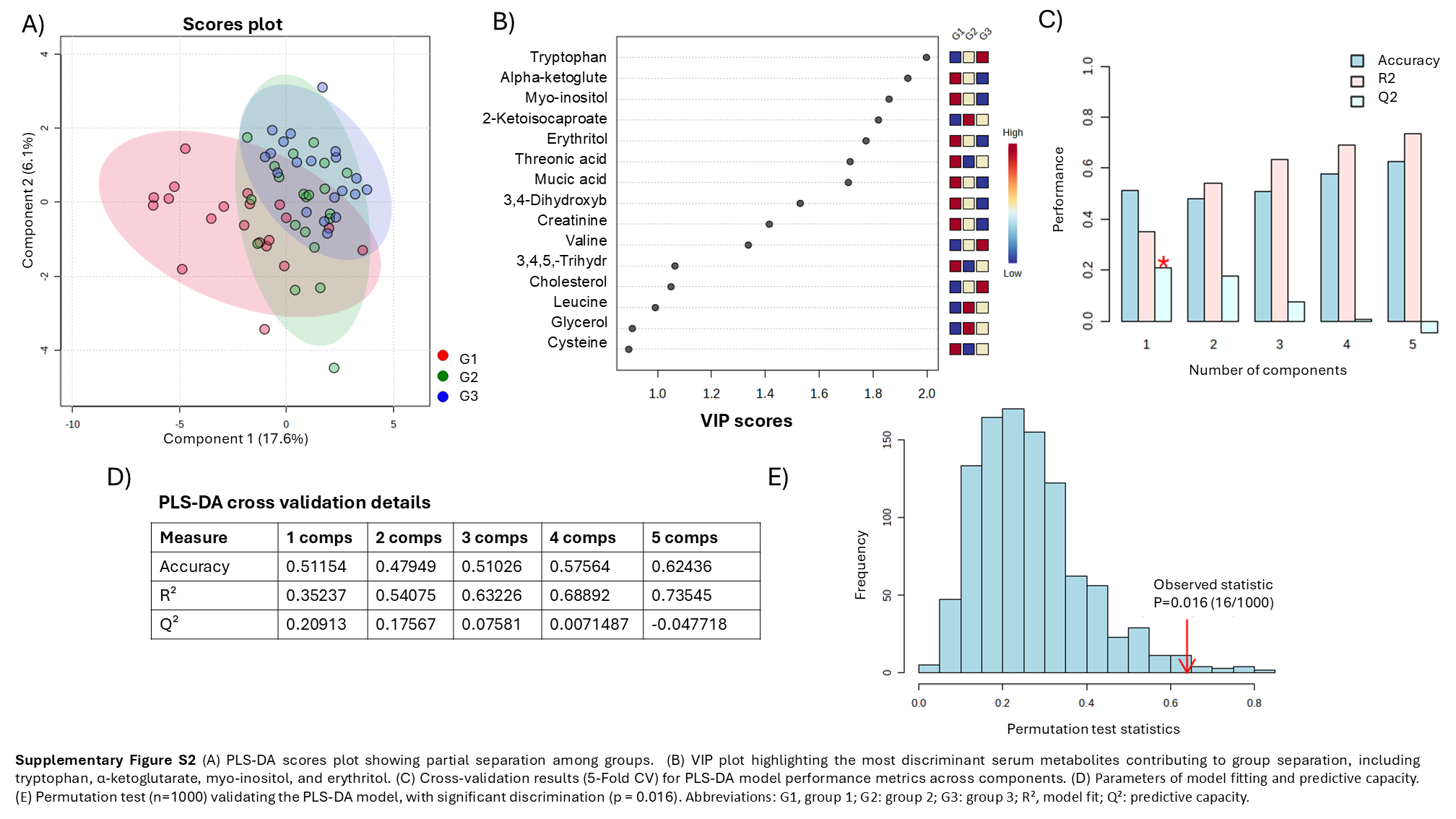

Supplement: Supplementary Table 2 — Serum metabolomic profile according to study group [file Image2.png]

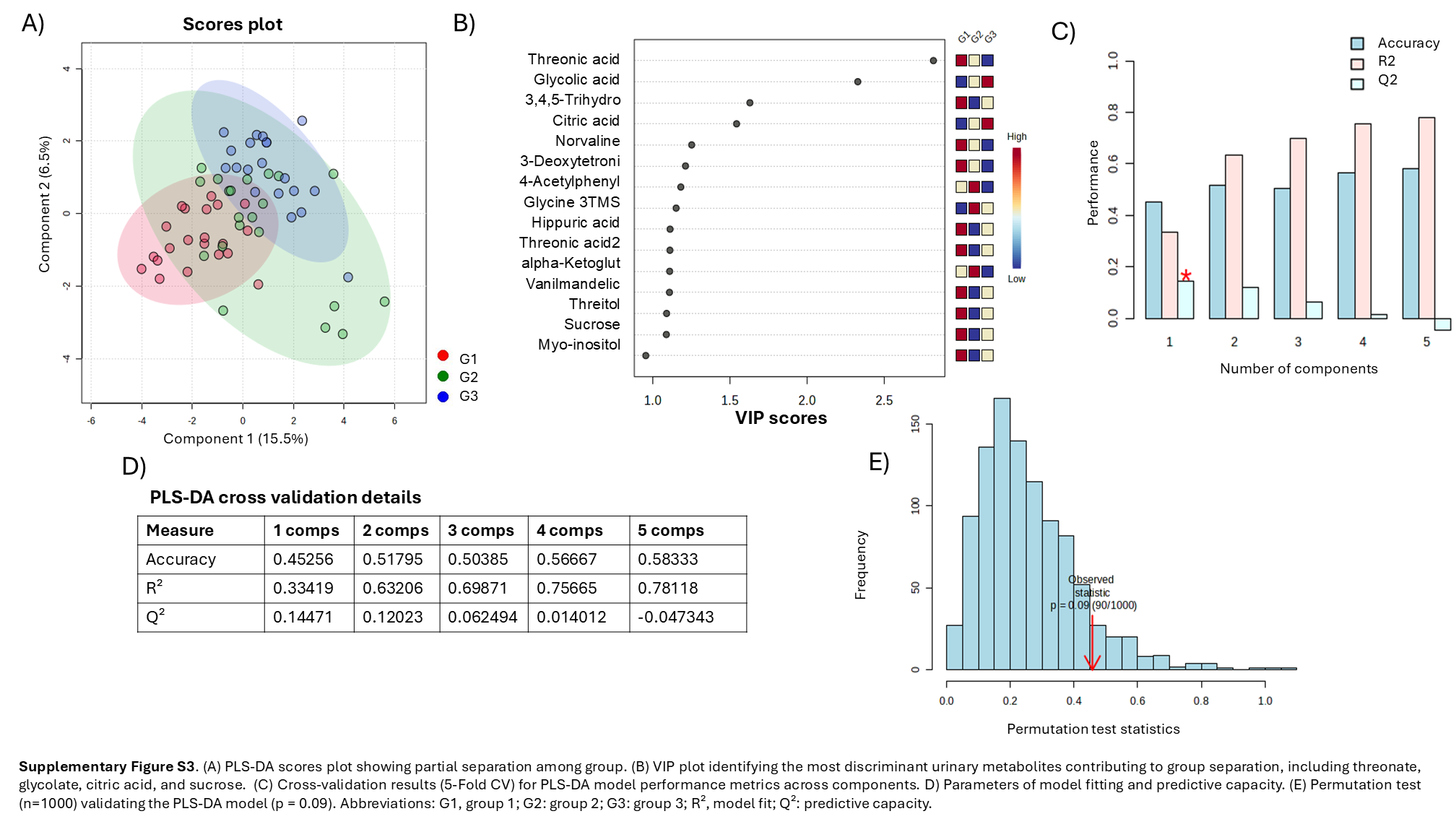

Supplement: Supplementary Table 3 — Urinary metabolomic profile according to study group. ++ Different retention time. [file Image3.png]
