# Supplementary material for: Integrated metabolomic and cytokine profiling reveals biomarkers across the clinical spectrum of lupus nephritis
Source: Front Immunol. 2026 Mar 31;17:1787160. doi: 10.3389/fimmu.2026.1787160 (PMC13076570; doi:10.3389/fimmu.2026.1787160)
Supplement: Supplementary Table 4 — Urine cytokines according to study groups. G-CSF, Granulocyte colony-stimulating factor; GM-CSF, Granulocyte-macrophage colony-stimulating factor; IFN-γ, Interferon-gamma; MCP-1, Monocyte chemoattractant protein-1; MIP-1b, Active Macrophage Inflammatory Protein 1 Beta; TNF-α, Tumor necrosis factor-alpha. FDR; False discovery rate. [file Table1.docx]

Supplementary material

Supplementary Table 1 S1. Therapeutic variables according to study group

| Variables | Active LN  Group 1  (n=21) | Post-induction responders  Group 2  (n=21) | Sustained responders  Group 3  (n=20) | p-value |
| --- | --- | --- | --- | --- |
| Steroid use  *n (%)* | 18 (86%) | 17 (81%) | 5 (25%) | <0.001 |
| Prednisone dose (mg/day)  *median (IQR)* | 10 (7.5, 28.8) | 5 (5, 5) | 5 (5, 5) | 0.002 |
| MMF  *n (%)* | 12 (57%) | 19 (90%) | 11 (55%) | 0.023 |
| MMF dose (gr/day)  *median (IQR)* | 2.25 (2, 2.6) | 2.5 (2, 2.7) | 1.5 (1, 2) | 0.006 |
| Azathioprine  *n (%)* | 4 (19%) | 0 | 5 (25%) | 0.041 |
| Cyclophosphamide  *n (%)* | 1 (4.8%) | 3 (14%) | 0 (0%) | 0.3 |
| Hydroxychloroquine  *n (%)* | 16 (76%) | 20 (95%) | 15 (75%) | 0.2 |
| Tacrolimus  *n (%)* | 4 (19%) | 8 (38%) | 0 (0%) | 0.006 |
| Rituximab  *n (%)* | 2 (9.5%) | 4 (19%) | 0 (0%) | 0.2 |
| ACE inhibitors/ARBs  *n (%)* | 17 (81%) | 19 (90%) | 12 (60%) | 0.073 |
| SGLT2 inhibitors  *n (%)* | 3 (14%) | 8 (38%) | 1 (5%) | 0.028 |
| Statin  *n (%)* | 4 (19%) | 4 (19%) | 2 (10%) | 0.8 |

Abbreviations: MMF, mycophenolate mofetil; ACE, angiotensin-converting enzyme; ARBs, angiotensin II receptor blockers; SGLT2, sodium-glucose transport protein 2 (SGLT2) inhibitors.

Supplementary Table 2 S2. Serum metabolomic profile according to study group

| Metabolite  Maximum peak heights (sum-normalized) | Overall (n=62)  *Median (IQR)* | Active LN  Group 1  (n=21)  *Median (IQR)* | Post-induction responders  Group 2  (n=21)  *Median (IQR)* | Sustained responders  Group 3  (n=20)  *Median (IQR)* | p-value | p-value FDR corrected |
| --- | --- | --- | --- | --- | --- | --- |
| 1,5-Anhydroglucitol | 56 (33, 71) | 54 (47, 72) | 47 (15, 69) | 58 (45, 74) | 0.4 | 0.01228 |
| 2-Aminobutyric acid | 11.5 (9.5, 14.8) | 10.5 (9.6, 15.6) | 12.2 (10.2, 14.8) | 10.0 (9.1, 13.9) | 0.5 | 0.6144 |
| 2-Ethyl-3-hydroxypropionic acid | 0.09 (0.06, 0.11) | 0.09 (0, 0.11) | 0.08 (0.07, 0.1) | 0.10 (0.07, 0.13) | 0.5 | 0.6182 |
| α-Ketoisocaproic acid | 6.02 (4.65, 7.01) | 4.45 (3.54, 5.51) | 6.80 (5.56, 7.45) | 6.40 (5.3, 7.7) | 0.001 | 0.6182 |
| 2-Deoxy-D-ribonic acid | 2.18 (1.58, 3.44) | 3.07 (2.21, 4.65) | 1.93 (1.50, 2.43) | 1.86 (1.51, 2.37) | 0.002 | 0.0137 |
| 4-Deoxy-erythronic acid | 1.67 (1.29, 2.18) | 2.38 (1.77, 3.39) | 1.39 (1.29, 1.89) | 1.39 (1.15, 1.72) | <0.001 | 0.01039 |
| 3-Hydroxybutyric acid | 23 (15, 31) | 20 (15, 25) | 27 (17, 39) | 21 (14, 29) | 0.2 | 0.4964 |
| 4-Hydroxyproline | 5.15 (4.06, 7.94) | 5.18 (4.49, 7.20) | 5.32 (4.07, 8.91) | 4.94 (3.67, 8.8) | >0.9 | 0.9289 |
| Alanine | 181 (162, 197) | 188 (179, 207) | 176 (159, 186) | 184 (160, 200) | 0.084 | 0.233 |
| α-Hydroxybutyric acid | 14 (10, 19) | 12 (7, 16) | 16 (12, 19) | 14 (10, 19) | 0.3 | 0.5764 |
| α-Hydroxyisovaleric acid | 3.30 (2.41, 4.27) | 3.35 (2.52, 3.90) | 3.00 (2.66, 3.87) | 3.45 (2.34, 4.53) | >0.9 | 0.9472 |
| α-Ketoglutaric acid | 3.52 (2.94, 4.02) | 3.97 (3.61, 4.59) | 3.18 (2.86, 4.04) | 3.25 (2.87, 3.61) | <0.001 | 0.01039 |
| Azelaic acid | 6.8 (2.4, 9.4) | 7.9 (6.2, 11.2) | 2.4 (0.0, 7.7) | 7.3 (5.9, 9.2) | 0.014 | 0.04976 |
| β-Aminoisobutyric acid | 0.81 (0.53, 1.53) | 0.98 (0.57, 2.65) | 0.70 (0.53, 1.09) | 0.86 (0.56, 1.69) | 0.5 | 0.6182 |
| Cholesterol | 31 (24, 41) | 36 (27, 47) | 28 (23, 35) | 29 (24, 34) | 0.2 | 0.4964 |
| Citrate | 6.67 (5.51, 9.05) | 6.10 (4.37, 8.56) | 6.57 (5.69, 7.71) | 7.87 (6.04, 9.67) | 0.4 | 0.6144 |
| Creatinine | 9.7 (8.4, 12.4) | 11.3 (8.9, 19.8) | 9.7 (8.4, 11.1) | 9.1 (8.3, 11.4) | 0.2 | 0.4143 |
| Cysteine | 3.54 (2.75, 4.10) | 3.96 (3.61, 4.63) | 3.00 (2.56, 3.56) | 3.47 (2.97, 4.01) | 0.005 | 0.0215 |
| Erythritol | 1.56 (1.13, 2.03) | 2.10 (1.55, 3.65) | 1.29 (1.07, 1.74) | 1.37 (1.06, 1.65) | <0.001 | 0.01039 |
| Glutamic acid | 11.0 (7.2, 14.5) | 11.6 (9.7, 14.7) | 11.0 (6.4, 13.6) | 10.6 (7.0, 14.6) | 0.4 | 0.6144 |
| Glutamine | 53 (47, 64) | 57 (44, 69) | 57 (47, 68) | 51 (49, 57) | 0.6 | 0.6795 |
| Glyceric acid | 7.61 (5.86, 8.79) | 7.41 (5.58, 8.27) | 7.87 (6.41, 10.25) | 7.56 (6.76, 9.00) | 0.5 | 0.6182 |
| Glycerol | 17 (12, 22) | 14 (11, 16) | 19 (18, 26) | 18 (13, 21) | 0.026 | 0.08509 |
| Glycolic acid | 4.13 (3.62, 5.08) | 4.17 (3.74, 4.83) | 3.92 (3.62, 4.99) | 4.30 (3.65, 5.57) | 0.7 | 0.7951 |
| Isoleucine | 45 (39, 51) | 45 (38, 49) | 45 (40, 53) | 45 (38, 51) | 0.8 | 0.857 |
| Leucine | 85 (74, 91) | 77 (66, 90) | 89 (80, 92) | 85 (77, 95) | 0.083 | 0.233 |
| Malic acid | 1.61 (1.26, 1.82) | 1.65 (1.39, 1.85) | 1.55 (1.31, 1.94) | 1.51 (1.22, 1.70) | 0.3 | 0.6088 |
| Methionine | 8.66 (7.79, 9.35) | 8.26 (7.28, 9.09) | 8.82 (7.97, 9.86) | 8.59 (8.00, 9.30) | 0.4 | 0.6144 |
| Galactaric acid | 1.34 (0.88, 2.14) | 2.39 (1.20, 3.70) | 1.28 (0.88, 1.85) | 1.11 (0.75, 1.62) | 0.011 | 0.04329 |
| Myo-inositol | 13 (11, 16) | 16 (13, 26) | 13 (11, 15) | 12 (10, 14) | 0.003 | 0.01481 |
| Palmitic acid | 45 (38, 55) | 45 (38, 49) | 53 (40, 66) | 41 (37, 55) | 0.2 | 0.4223 |
| Phenylalanine | 25.7 (23.6, 28.6) | 25.8 (24.5, 29.3) | 25.6 (21.8, 28.8) | 25.5 (24.6, 27.7) | 0.5 | 0.6182 |
| Serine | 60 (52, 64) | 60 (52, 65) | 61 (57, 66) | 58 (49, 62) | 0.4 | 0.6144 |
| Threonic acid | 3.51 (2.88, 5.31) | 5.20 (3.47, 7.45) | 3.22 (2.84, 3.79) | 3.36 (2.49, 4.49) | 0.003 | 0.01481 |
| Threonic acid (form 2) | 5.3 (3.4, 7.0) | 4.4 (3.1, 5.9) | 5.5 (3.1, 7.0) | 5.7 (3.9, 7.1) | 0.6 | 0.6856 |
| Threonine | 45 (39, 53) | 43 (40, 48) | 42 (36, 47) | 47 (42, 55) | 0.4 | 0.6144 |
| Tryptophan | 60 (50, 70) | 51 (37, 61) | 60 (57, 66) | 70 (58, 75) | 0.002 | 0.0137 |
| Tyrosine | 60 (52, 68) | 59 (50, 67) | 56 (51, 67) | 64 (55, 70) | 0.4 | 0.6144 |
| Valine | 57 (53, 62) | 55 (50, 58) | 57 (55, 61) | 58 (55, 64) | 0.10 | 0.2521 |

Supplementary Table 3 S3. Urinary metabolomic profile according to study group.

| Metabolite  Maximum peak heights (sum-normalized) | Overall (n=62) | Active LN  Group 1  (n=21)  *Median (IQR)* | Post-induction responders  Group 2  (n=21)  *Median (IQR)* | Sustained responders  Group 3  (n=20)  *Median (IQR)* | p-value | p-value FDR  corrected |
| --- | --- | --- | --- | --- | --- | --- |
| 2,3-dihydroxybutanoic acid | 6.1 (4.7, 9.2) | 7.2 (5.0, 9.0) | 5.1 (3.7, 6.1) | 7.2 (5.5, 10.5) | 0.037 | 0.1323 |
| 3,4,5-trihydroxypentanoic acid | 48 (39, 65) | 58 (45, 79) | 46 (38, 55) | 46 (39, 57) | 0.026 | 0.1098 |
| 3,4-dihydroxybutanoic acid | 23 (17, 30) | 23 (19, 34) | 23 (16, 29) | 24 (17, 27) | 0.7 | 0.847 |
| 3-deoxytetronic acid | 5.74 (5.18, 7.72) | 6.79 (5.59, 8.97) | 5.37 (4.97, 7.01) | 5.52 (4.78, 6.44) | 0.061 | 0.168 |
| 3-hydroxyisobutyric acid | 11 (8, 15) | 8 (8, 11) | 12 (9, 14) | 12 (7, 16) | 0.2 | 0.3586 |
| 4-acetylphenyl-beta-d-glucopyranosiduronic acid | 0.52 (0.20, 0.94) | 0.60 (0.34, 0.96) | 0.65 (0.25, 1.05) | 0.25 (0.09, 0.58) | 0.066 | 0.168 |
| 4-nitrophenyl-beta-d-galacturonide | 5 (3, 7) | 5 (4, 7) | 4 (2, 5) | 5 (4, 10) | 0.041 | 0.14 |
| 5-methyluridine | 16 (10, 25) | 20 (11, 29) | 16 (10, 20) | 12 (7, 24) | 0.6 | 0.7494 |
| 6-hydroxy-alpha-methylnaphtaleneacetic acid | 5.24 (4.07, 6.71) | 6.15 (5.09, 6.78) | 5.02 (4.06, 6.65) | 4.55 (4.03, 5.68) | 0.2 | 0.3586 |
| Acetaminophen | 20 (12, 27) | 20 (12, 34) | 15 (12, 21) | 21 (15, 26) | 0.4 | 0.5946 |
| Alanine | 28 (19, 38) | 29 (20, 47) | 27 (19, 39) | 28 (17, 35) | 0.8 | 0.8811 |
| Alpha-hydroxybutyric acid | 4.12 (3.05, 6.62) | 5.58 (3.60, 7.55) | 3.82 (2.94, 6.64) | 3.66 (3.16, 5.07) | 0.3 | 0.4226 |
| Alpha-hydroxyisobutyric acid | 4.17 (3.29, 5.44) | 4.05 (2.66, 4.86) | 4.63 (3.74, 5.91) | 4.05 (3.25, 5.06) | 0.2 | 0.3586 |
| Alpha-ketoglutaric acid | 24 (17, 28) | 25 (20, 28) | 23 (17, 47) | 21 (15, 26) | 0.2 | 0.3619 |
| Benzyl alcohol | 10.3 (8.2, 14.6) | 11.1 (8.2, 15.2) | 9.6 (8.3, 13.5) | 10.2 (8.7, 14.8) | 0.8 | 0.8811 |
| Citric acid | 25 (6, 53) | 9 (5, 25) | 27 (4, 41) | 53 (25, 66) | 0.008 | 0.0944 |
| Creatinine | 235 (161, 271) | 222 (165, 277) | 248 (212, 269) | 213 (155, 262) | 0.9 | 0.8811 |
| Cysteine | 4.11 (3.31, 5.90) | 4.12 (3.00, 5.62) | 3.58 (3.28, 6.54) | 4.66 (3.83, 5.47) | 0.8 | 0.8811 |
| Gluconic acid | 0.29 (0.21, 0.39) | 0.29 (0.21, 0.38) | 0.28 (0.20, 0.46) | 0.29 (0.23, 0.37) | >0.9 | 0.9432 |
| Glycine | 50 (29, 91) | 31 (22, 65) | 60 (41, 115) | 48 (34, 100) | 0.064 | 0.168 |
| Glycolic acid | 46 (27, 78) | 31 (19, 42) | 48 (35, 85) | 71 (35, 89) | 0.002 | 0.02907 |
| Hippuric acid derivative | 9 (6, 21) | 14 (7, 25) | 9 (6, 13) | 7 (5, 15) | 0.2 | 0.3484 |
| Lactose | 11 (7, 16) | 11 (8, 16) | 14 (10, 17) | 8 (6, 12) | 0.2 | 0.3586 |
| Levoglucosan | 20 (9, 37) | 13 (9, 27) | 19 (10, 46) | 23 (10, 38) | 0.6 | 0.7494 |
| Myo-inositol | 4 (3, 11) | 5 (4, 17) | 4 (2, 12) | 4 (3, 6) | 0.2 | 0.3586 |
| N-acetyl-d-glucosamine | 8.7 (7.1, 10.5) | 9.1 (7.8, 11.5) | 7.8 (6.5, 9.9) | 8.9 (6.5, 10.6) | 0.2 | 0.3586 |
| Norvaline | 4.87 (4.10, 5.74) | 5.57 (4.86, 6.21) | 4.84 (3.86, 5.64) | 4.49 (3.28, 4.94) | 0.025 | 0.1098 |
| P-cresol | 11 (5, 32) | 10 (3, 27) | 12 (6, 34) | 11 (5, 44) | 0.5 | 0.69 |
| P-tolyl-beta-d-glucuronide | 10 (5, 16) | 10 (7, 21) | 7 (4, 12) | 12 (6, 19) | 0.12 | 0.2803 |
| Pyruvic/lactic acid | 17 (12, 28) | 17 (14, 22) | 15 (10, 29) | 17 (12, 29) | 0.7 | 0.847 |
| Ribonic acid | 14.9 (11.4, 17.3) | 16.3 (15.0, 18.1) | 14.3 (10.2, 16.1) | 11.9 (10.8, 16.7) | 0.023 | 0.1098 |
| Sucrose | 10 (5, 23) | 15 (9, 44) | 6 (4, 14) | 12 (6, 16) | 0.017 | 0.1098 |
| Threitol | 24 (19, 29) | 29 (21, 38) | 21 (17, 24) | 24 (18, 28) | 0.055 | 0.168 |
| Threonic acid | 94 (78, 120) | 125 (103, 133) | 89 (69, 106) | 82 (76, 93) | <0.001 | 0.0008712 |
| Threonic acid^++^ | 21.7 (18.2, 25.0) | 23.7 (22.2, 27.1) | 19.0 (15.6, 23.9) | 20.3 (18.0, 22.6) | 0.013 | 0.1003 |
| Urea | 22 (18, 28) | 22 (19, 29) | 20 (16, 26) | 22 (19, 30) | 0.4 | 0.6327 |
| Vanilmandelic acid | 4.71 (2.95, 5.73) | 5.43 (4.84, 7.76) | 4.62 (0.31, 5.64) | 4.11 (2.73, 4.94) | 0.010 | 0.0944 |
| Xhantine | 3.9 (2.3, 5.8) | 3.9 (3.0, 6.4) | 3.8 (1.7, 6.0) | 3.4 (2.7, 5.1) | 0.7 | 0.847 |

^++^ Different retention time.

Supplementary Table 4 S4. Urine cytokines according to study groups.

| Urinary cytokines  ( **pg/mL**) | Active LN  Group 1  (n=21)  *Median (IQR)* | Post-induction responders  Group 2  (n=21)  *Median (IQR)* | Sustained responders  Group 3  (n=20)  *Median (IQR)* | p-value | p-value FDR corrected |
| --- | --- | --- | --- | --- | --- |
| IL-1β | 0.42 (0.08, 1.10) | 0.08 (0, 0.31) | 0 | <0.001 | 0.0002229 |
| IL-2 | 0.75 (0.50, 1.00) | 0.24 (0, 0.24) | 0 (0, 0.24) | <0.001 | 1.634 x10^-05^ |
| IL-4 | 0 (0, 0.05) | 0 (0, 0.05) | 0 | 0.2 | 0.1767 |
| IL-5 | 3.41 (3.41, 6.52) | 0.90 (0, 3.41) | 0 | <0.001 | 3.698 x10^-05^ |
| IL-6 | 3.2 (2.1, 9.7) | 0.4 (0.3, 1.1) | 0.3 (0.2, 0.4) | <0.001 | 1.355 x10^-06^ |
| IL-7 | 0.65 (0, 2.57) | 0 | 0 | 0.001 | 1.167 x10^-05^ |
| IL-8 | 9 (5, 26) | 4 (1, 5) | 1 (0, 4) | <0.001 | 0.0002229 |
| IL-10 | 0.75 (0.60, 0.90) | 0.30 (0.30, 0.60) | 0.30 (0.01, 0.30) | <0.001 | 8.429 x10^-06^ |
| IL-12 | 0 (0, 0.66) | 0 | 0 | 0.4 | 0.14 |
| IL-13 | 0.08 (0.08, 0.08) | 0 (0, 0.08) | 0 (0, 0.04) | <0.001 | 0.0002229 |
| IL-17 | 0.26 (0.15, 0.38) | 0.15 (0, 0.15) | 0.15 (0, 0.15) | 0.023 | 0.02613 |
| G-CSF | 7.3 (4.7, 9.8) | 3.4 (0, 4.7) | 0 (0, 1.1) | <0.001 | 1.572 x10^-06^ |
| GM-CSF | 0.43 (0, 0.74) | 0 | 0 | <0.001 | 1.572 x10^-06^ |
| IFN-γ | 1.31 (0.44, 3.23) | 0.26 (0.26, 0.44) | 0.26 (0.09, 0.37) | <0.001 | 7.828 x10^-06^ |
| MCP-1 | 131 (39, 388) | 6 (4, 16) | 4 (3, 10) | <0.001 | 1.355 x10^-06^ |
| MIP-1b | 3.9 (2.9, 11.8) | 1.1 (0.8, 1.7) | 0.5 (0.1, 0.9) | <0.001 | 1.632 x10^-06^ |
| TNF-α | 1.24 (0, 4.24) | 0 | 0 | 0.002 | 2.993 x10^-06^ |

Abbreviations: G-CSF, Granulocyte colony-stimulating factor; GM-CSF, Granulocyte-macrophage colony-stimulating factor; IFN-γ, Interferon-gamma; MCP-1, Monocyte chemoattractant protein-1; MIP-1b, Active Macrophage Inflammatory Protein 1 Beta; TNF-α, Tumor necrosis factor-alpha. FDR; False discovery rate.
